# Pulmonary artery catheter placement in patients with a history of tricuspid ring annuloplasty, with pulmonary stenosis, and with the transvenous pacemaker leads: is it difficult?

**DOI:** 10.1186/s40981-018-0163-4

**Published:** 2018-03-13

**Authors:** Hirofumi Hamaba, Yuka Miyata, Yukio Hayashi

**Affiliations:** Anesthesiology Service, Sakurabashi-Watanabe Hospital, 2-4-32 Umeda, Kita-ku, Osaka, 530-0001 Japan

**Keywords:** Pulmonary artery catheter, Difficult placement, Tricuspid ring annuloplasty, Transvenous pacemaker leads

## Abstract

**Purpose:**

Previous case reports suggested that the presence of obstruction in the passage of the pulmonary artery catheter (PAC) may hinder its placement. However, little is known regarding the difficulty in proper placement of the PAC in those patients. This prospective observational study was designed to examine the difficulty in PAC placement in the patients with a possible obstruction in the passage of the PAC, and compare it with that of patients without those complications.

**Method:**

We measured the time required for placement of the PAC in adult patients with a history of tricuspid ring annuloplasty, with pulmonary artery stenosis or with transvenous pacemaker leads, and in patients without those history or complications (control). The flow-directed balloon-tipped PAC was placed under monitoring of pulmonary artery pressure wave. If the PAC was placed within 5 min, we regarded it was successful. We compared the success rate and the PAC placement time among the four groups of patients.

**Results:**

The number of patients with a history of tricuspid ring annuloplasty, with pulmonary artery stenosis, with transvenous pacemaker leads and the control was 4, 1, 16, and 379, respectively. Placement of the PAC was successful in only one of the four patients with a history of tricuspid ring annuloplasty, and the success rate was significantly lower than the control patients (*P* < 0.001). We could finally place the PAC into the pulmonary artery under X-ray fluoroscopic photographing system in the other three patients. On the other hand, the PAC was successfully inserted in all patients with transvenous pacemaker leads and with pulmonary artery stenosis.

**Conclusion:**

Placement of a PAC under monitoring the pulmonary artery pressure waveform was difficult in a patient with history of tricuspid ring annuloplasty, but not in patients with transvenous pacemaker leads.

## Introduction

The pulmonary artery catheter (PAC) is used for perioperative management in patients undergoing cardiovascular surgery, although its application still remained controversial [1–3]. We routinely place the catheter in patients undergoing cardiovascular surgery after induction of anesthesia by monitoring the pressure waveform. The flow-directed balloon-tipped PAC is readily carried to the pulmonary artery (PA). However, in some cases, we fail its placement by simply observing the PA pressure wave. The difficulty of the catheter placement may, in part, depend on operator’s skill but to a large extent be due to patient-related factors. One may claim that the presence of obstruction in the passage of the PAC such as history of tricuspid ring annuloplasty, pulmonary stenosis, and the transvenous pacemaker leads may hinder the catheter placement. However, little has been written regarding difficulties in achieving proper placement of PAC in patients with these factors except three case reports [4–6]. For recent 3 years (2014, November ~  2017, October), we placed the PAC in 400 patients undergoing cardiovascular surgery after anesthesia and encountered 21 patients with one of these factors in our hospital. This prospective observational study was designed to compare difficulties during the PAC placement in the patients having a factor above mentioned with patients without any of these factors.

## Method

The current study was approved by the institutional review board, and informed consent was obtained from all eligible patients. It was registered in the UMIN Clinical Trial Registry (UMIN000030025). This study was conducted from November 2014 to October 2017 at Sakurabashi-Watanabe hospital in Osaka, Japan. We prospectively examined the time required for the PAC placement in 400 adult patients undergoing elective cardiovascular surgery. All patients were monitored electrocardiogram, invasive arterial pressure, oxygen saturation, and end-tidal carbon dioxide. After induction of anesthesia with midazolam, fentanyl or remifentanil, and vecuronium, mechanical ventilation was started following tracheal intubation. Anesthesia was maintained with propofol or sevoflurane combined with remifentanil and fentanyl. The PAC (continuous cardiac output/SvO_2_ Catheter 744HF75, Edwards Lifesciences, Irvine, CA, USA) was inserted through the right internal jugular vein. First, the introducer sheath was placed via the right internal jugular vein in the Trendelenburg position, and then the PAC was started floating through the sheath by monitoring the pressure waveform in the flat position. The PAC was inserted approximately 20 cm, and central venous pressure (CVP) waveform was confirmed, subsequently the balloon was inflated with 1.5 ml of air. With inflated balloon, the catheter was floated into the PA. The waveform of the PA was first observed followed by inserting the catheter approximately 2–3 cm forward and deflated the balloon. In this position, after confirming that the tip of the catheter was not wedged into the PA, it was locked with the sheath.

The time required for placement of a PAC was measured. The catheter placement time was defined as the duration required for the catheter to float from the CVP position through the right heart chambers to the PA, that is, the beginning time point was just after the inflation of the balloon to start floating the catheter and the ending time point is the time which we first observed the waveform of the PA [7]. If the placement was done within 5 min, we regarded this case as successful. On the other hand, if the placement failed to precede the catheter into the PA in 5 min, we regarded this case as failure and some guidance such as transesophageal echocardiography or X-ray fluoroscopic system to visualize intracardiac catheter orientation was used.

In this study, we collected the data in patients with history of tricuspid ring annuloplasty, pulmonary stenosis, or transvenous pacemaker leads and compared with those in patients without any of them (control).

Before the study started, we planned to collect 400 cases and then to perform sample size calculation. As a result, we had available results in these 400 cases and waived power of analysis. The successful rate of the PAC placement was expressed in percentages and was analyzed by Fisher’s exact test followed by a Bonferroni multiple comparisons to specify differences between groups. The placement times were expressed as median and interquartile range and analyzed by Kruskal-Wallis test followed by Mann-Whitney test with Bonferroni multiple comparisons to specify differences between groups. The statistical analysis was performed by SPSS (IBM Corporation, USA) version 14.0. *P* < 0.05 was considered statically significant.

## Results

There were 4 cases with history of tricuspid ring annuloplasty, 1 case with pulmonary artery stenosis, and 16 cases with the transvenous pacemaker leads. The number of the subject without any of those history or complications (control) was 379. The successful rate and PAC placement time in each category is shown in Table 1. The placement was significantly difficult in patients with history of tricuspid ring annuloplasty. It was successful only in one patient, and the success rate was significantly lower in patients with a history of tricuspid ring annuloplasty compared with the control (*P* < 0.001). We placed the PAC under X-ray fluoroscopic photographing system in other three cases. On the other hand, the placement in patients with transvenous pacemaker leads was not significantly different from that of the control. We encountered only one patient with pulmonary artery stenosis. He was a 70-year-old male and was scheduled for aortic valve replacement. He was diagnosed as tetralogy of Fallot at birth and underwent radical operation at 29 years. He had residual systolic pressure gradient of 30 mmHg between the pulmonary artery and the right ventricle (Table 2).

**Table 1:** Patients’ characteristics in each category

	Age (years)	Height (cm)	Weight (kg)	BSA (m^2^)	Diseases (valve/coronary/aorta/valve+coronary/congenital/others)
Tricuspid ring annuloplasty	61 ± 15	157 ± 7	51 ± 7	1.5 ± 0.1	4/0/0/0/0/0
Pulmonary artery stenosis	70	155	47	1.5	1/0/0/0/0/0
Transvenous pacemaker leads	74 ± 5	158 ± 8	59 ± 8	1.6 ± 0.2	13/0/1/2/0/0/0
Control	68 ± 12	161 ± 10	60 ± 12	1.6 ± 0.2	199/77/64/20/6/13

Control means subjects without any factor above mentioned

Data were expressed as means ± SD

**Table 2:** Successful rate and the PAC placement time in each category

	*N* (success/failure)	Successful rate (%)	Placement time (s)
Tricuspid ring annuloplasty	4 (1/3)	25*	283*
Pulmonary artery stenosis	1 (1/0)	100	25
Transvenous pacemaker leads	16 (16/0)	100	28 (17 ~ 97)
Control	379 (374/5)	98.7	25 (17 ~ 50)

Control means subjects without any factor abovementioned

Placement time is given as median (interquartile range)

*P < 0.05 vs control

## Discussion

The present data reconfirmed that placement of a PAC is difficult in patients with history of tricuspid ring annuloplasty. On the other hand, placement of a PAC by monitoring the pressure waveform is possible in patients with the transvenous pacemaker leads.

According to the previous case reports [5, 6], one may consider that placement of a PAC may be difficult in patients with history of tricuspid ring annuloplasty. However, to our knowledge, there has been no clinical study to examine whether the placement in patients with history of tricuspid ring annuloplasty is difficult or not in comparison with patients without this history. Although we could encounter only four cases during the observation period, we could reconfirm that the placement was significantly difficult compared with the control. Finally, we could place the catheter under X-ray fluoroscopic photographing system. Since the tricuspid ring clearly appears in the X-ray system, it is a helpful maker to facilitate the catheter to pass the tricuspid valve. Thus, we guess that we should not hesitate to prepare the X-ray system in advance. Presumably, this conclusion seems to be acceptable for anesthesiologists.

One previous case report suggested that transvenous pacemaker leads may be a possible obstacle to hinder the PAC placement [4]. However, we had a clinical impression that the transvenous pacemaker lead is not a significant problem for the placement. In this study, we encountered 16 patients with transvenous pacemaker leads during the study period. As shown in Table 1, we could successfully place the catheter by monitoring the pressure waveform in all subjects and the placement tine was similar to that of the control group. Thus, although one might claim that the number of subject in this study is small, we suggest that the catheter placement can be done by monitoring the pressure waveform in patients with transvenous pacemaker leads and some guidance such as X-ray fluoroscopic photographing system may not be prepared in advance.

Unfortunately, we could encounter only one case with pulmonary stenosis. Thus, we cannot conclude whether the PAC placement is possible by monitoring the pressure waveform. However, based on our experience of this one case, we have an impression that we are worth placing the catheter with the pressure monitoring in a patient with pulmonary stenosis at first. With future accumulating the similar cases, a conclusion will be provided.

We have to discuss potential limitations in our study. Although we collected the subjects for 3 years, the number of patients having obstruction in the passage of the PAC was small. Our results are dependent on the data we accumulated during the study period, so the clinical significance of our results would be interpreted with caution. We have to acknowledge the possibility that accumulation of further number of subjects would change our conclusion in the future.

In conclusion, placement of a PAC by monitoring the pressure waveform is difficult in a patient with history of tricuspid ring annuloplasty and the placement under X-ray fluoroscopic photographing system is recommended. On the other hand, the placement by monitoring the pressure waveform may be available in a patient with transvenous pacemaker leads.
